# Crystal structure of a mixed-ligand dinuclear Ba—Zn complex with 2-meth­oxy­ethanol having tri­phenyl­acetate and chloride bridges

**DOI:** 10.1107/S2056989015011226

**Published:** 2015-06-17

**Authors:** Józef Utko, Maria Sobocińska, Danuta Dobrzyńska, Tadeusz Lis

**Affiliations:** a Faculty of Chemistry, University of Wrocław, 14 Joliot-Curie St, 50-383 Wrocław, Poland; b Faculty of Chemistry, Wrocław University of Technology, 27 Wybrzeże Wyspiańskiego, 50-370 Wrocław, Poland

**Keywords:** crystal structure, Ba–Zn dinuclear complex, tri­phenyl­acetate ligand, 2-meth­oxy­ethanol, hydrogen bonding

## Abstract

The dinuclear barium–zinc complex, μ-chlorido-1:2κ^2^ *Cl*:*Cl*-chlorido-2κ*Cl*-bis­(2-meth­oxy­ethanol-1κ*O*)bis­(2-meth­oxy­ethanol-1κ^2^ *O*,*O*′)bis­(μ-tri­phenyl­acetato-1:2κ^2^ *O*:*O*′)bariumzinc, [BaZn(C_20_H_15_O_2_)_2_Cl_2_(C_3_H_8_O_2_)_4_], has been synthesized by the reaction of barium tri­phenyl­acetate, anhydrous zinc chloride and 2-meth­oxy­ethanol in the presence of toluene. The barium and zinc metal cations in the dinuclear complex are linked *via* one chloride anion and carboxyl­ate O atoms of the tri­phenyl­acetate ligands, giving a Ba⋯Zn separation of 3.9335 (11) Å. The irregular nine-coordinate BaO_8_Cl coordination centres comprise eight O-atom donors, six of them from 2-meth­oxy­ethanol ligands (four from two bidentate *O*,*O*′-chelate inter­actions and two from monodentate inter­actions), two from bridging tri­phenyl­acetate ligands and one from a bridging Cl donor. The distorted tetra­hedral coordination sphere of zinc comprises two O-atom donors from the tri­phenyl­acetate ligands and two Cl donors (one bridging and one terminal). In the crystal, O—H⋯Cl, O—H⋯O and C—H⋯Cl inter­molecular inter­actions form a layered structure, lying parallel to (001).

## Abstract 2

In a new Ba–Zn dimeric coordination complex which has 2-meth­oxy­ethanol as well as tri­phenyl­acetate and chlorido ligands, the BaO_8_Cl and ZnO_2_Cl_2_ complex centres are separated by 3.9335 (1) Å and are connected through two carboxyl *O,O^1^* bridges and one bridging chloride anion.

## Chemical context   

Only a few polynuclear heterometallic compounds containing barium and zinc connected by carboxyl­ate bridges are known (Akine *et al.*, 2006 ▸, 2009 ▸, 2010 ▸; Zhang *et al.*, 2012 ▸; Bo *et al.*, 2013 ▸). We have been studying the reactions of the tri­phenyl­acetate anion with metal salts and we have obtained several anhydrous polynuclear Mn^II^ tri­phenyl­acetate-containing clusters (Utko *et al.*, 2014 ▸). The complexes with some metals (for example: Fe, Ni, Cu, Ru, Rh, Ag) are reported in the literature (Yamanaka *et al.*, 1993 ▸; Cotton *et al.*, 1994 ▸; Akhbari & Morsali, 2010 ▸; Barberis *et al.*, 2001 ▸; Cadiou *et al.*, 2002 ▸; Do & Lippard, 2011 ▸). However, among polynuclear complexes with tri­phenyl­acetate ligands, dinuclear Ba–Zn representatives have not previously been reported. In the present work, we aimed to create a mixed-ligand compound containing zinc and barium cations, using barium tri­phenyl­acetate as a means of displacing chlorine atoms from zinc chloride. This procedure for removal of chlorine using tri­phenyl­acetate was successfully carried out in a reaction leading to the formation of a mixed-metal complex with a [Ba_4_Ti_2_] core (Kosińska-Klähn *et al.*, 2014 ▸). In the present paper we report the synthesis and structural characterization of a dinuclear Ba–Zn complex, namely μ-chlorido-1:2κ^2^ *Cl*:*Cl*-chlorido-2κ*Cl*-bis­(2-meth­oxyethanol-1κ*O*)bis­(2-meth­oxy­ethanol-1κ^2^ *O*,*O*′)bis­(μ-tri­phenylacetato-1:2κ^2^ *O*:*O*′)bariumzinc, (I), and the structure is discussed herein.
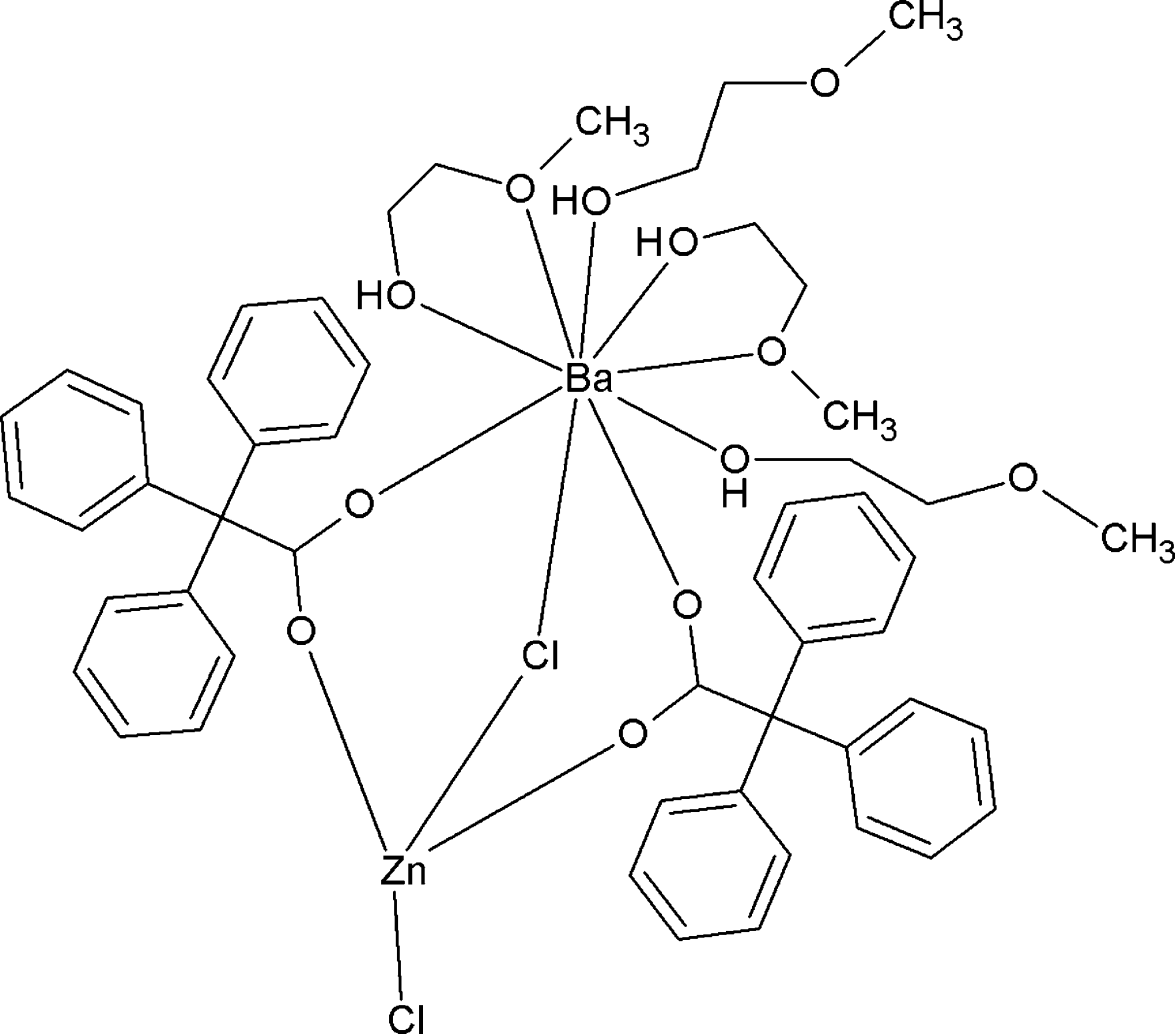


## Structural commentary   

**Figure 1:**
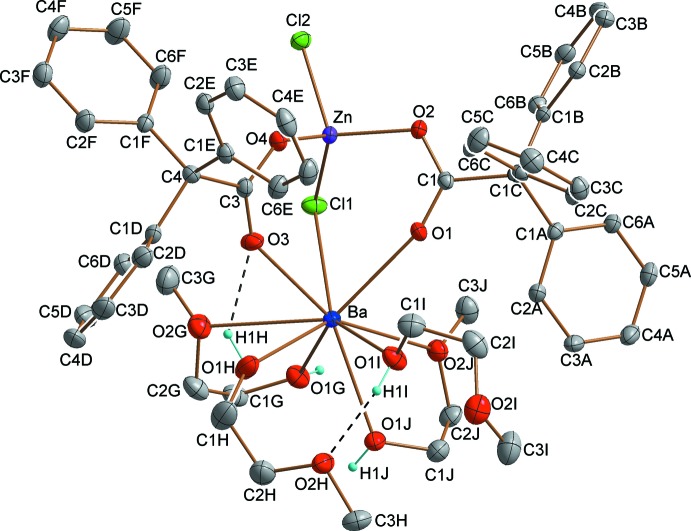
The mol­ecular structure of the title complex, with displacement ellipsoids drawn at the 50% probability level. Dashed lines represent intra-complex hydrogen bonds. C-bonded H atoms have been omitted for clarity.

In the structure of (I), the asymmetric unit contains one dinuclear complex of [BaZn(Ph_3_CCOO)_2_(CH_3_OCH_2_CH_2_OH)_4_Cl_2_] (Fig. 1 ▸), in which the dinuclear [BaZn]^4+^ cationic core is bridged by two carboxyl­ate arms of the tri­phenyl­acetate ligands in a κ^1^:κ^1^:μ^2^ coordination mode and by one bridging chlorine atom (μ_2_-Cl). The Ba⋯Zn distance in the dinuclear complex is 3.9335 (11) Å. Oxygen atoms have the largest contribution to the filling of the coordination sphere of barium [Ba—O bond-length range, 2.6925 (19)– 2.985 (2) Å; Table 1 ▸]. Barium is bonded to one bridging chloride atom (μ_2_-Cl), two O-atoms of two carboxyl­ate groups and also to six O atoms from the 2-meth­oxy­ethanol ligands (four from two bidentate *O*,*O* ^1^-chelate inter­actions and two from monodentate inter­actions). 2-Meth­oxy­ethanol is coordinated only to the Ba^2+^ cation. The coordination mode is achieved in two different ways. Two terminal mol­ecules representing an κ^1^:κ^1^ mode form two five-membered rings completed by the barium atom. Two other mol­ecules of 2-meth­oxy­ethanol coordinate to Ba only through the hydroxyl O atoms.

Zinc is four-coordinated with a distorted tetra­hedral ZnO_2_Cl_2_ stereochemistry (Table 1 ▸), with Zn—Cl1 (bridging) = 2.2595 (10) Å and Zn—Cl2 (monodentate) = 2.2653 (9) Å and Zn—O (both from the bridging tri­phenyl­acetate groups = 1.96817 (2) and 1.9683 (18) Å). A comparison with other structurally characterized mixed-metallic zinc–barium complexes reveals that the Zn–Cl–Ba linkage has been observed for the first time in the present compound. There are only a few compounds containing both of these metals and only one is a dimeric structure, with a distance between the atoms of 3.629 (2) Å, significantly shorter than in the title complex [3.9335 (11) Å], but zinc and barium are connected only *via* bridging oxygen atoms (μ_2_-O) from organic ligands (Van Veggel *et al.*, 1989 ▸). Also, in other structures without carboxyl­ate bridges, the Zn⋯Ba distances are often much shorter than in the title complex with values in the range 3.4325 (5) to 4.850 (3) Å (Westerhausen *et al.*, 2001 ▸, 2006 ▸; Baggio *et al.*, 2004 ▸; John *et al.*, 2008 ▸). In those cases where the oxygen atom (μ_2_-O) and also carboxyl­ates connect zinc and barium, the Zn⋯Ba distance is not longer than 3.638 (1) Å (Akine *et al.*, 2006 ▸, 2009 ▸, 2010 ▸). In a polymeric structure where zinc and barium cations are bridged *via* two carboxyl­ate arms and also *via* one mol­ecule of water, the distance between them is 4.0208 (5) Å (Zhang *et al.*, 2012 ▸).

## Supra­molecular features   

**Figure 2:**
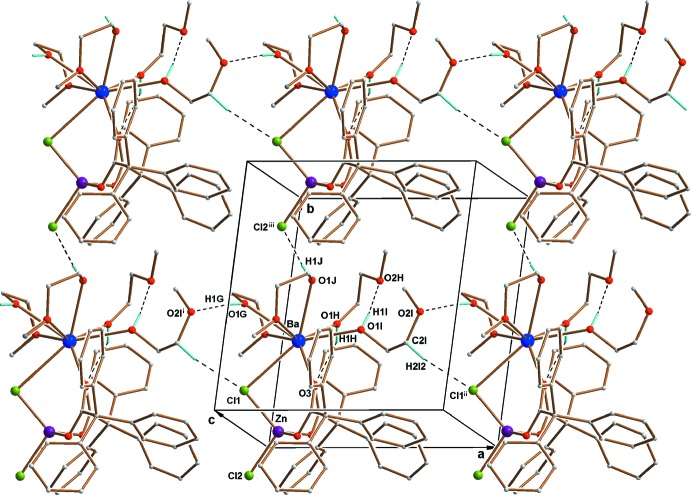
Part of the crystal structure of the complex. Dashed lines represent intra- and inter­molecular hydrogen bonds. C-bonded H atoms not involved in hydrogen bonding have been omitted for clarity. For symmetry codes, see Table 2 ▸.

In the crystal, there are intra­molecular O—H⋯O hydrogen bonds (Table 2 ▸). One is formed between a hydroxyl group O1*I* and an O-atom acceptor from the ether atom (O2*H*) of a 2-meth­oxy­ethanol ligand, the second is formed between a hydroxyl group O1*H* and an O-atom acceptor from a carboxyl group (O3) of a Ph_3_CCOO^−^ ligand (Fig. 1 ▸). The presence of electronegative atoms (oxygen and chlorine) also leads to the occurrence of inter­molecular hydrogen bonds in the crystal structure. The neighbouring dinuclear mol­ecules inter­act through O—H⋯O, O—H⋯Cl and C—H⋯Cl hydrogen bonds. The first one occurs between the hydroxyl group O1*G* and an ether O-atom acceptor O2*I* ^i^, the second occurs between the hydroxyl group O1*J* and the terminal chlorine atom Cl2^iii^. In the third inter­action, the H-donor atom is from a 2-meth­oxy­ethanol carbon (C2*I*), with the bridging chlorine atom (Cl1*I*)^ii^ acting as the H-atom acceptor (for symmetry codes, see Table 2 ▸). A two-dimensional network structure is generated (Fig. 2 ▸), lying parallel to (001).

## Synthesis and crystallization   

For the preparation of Ba(Ph_3_CCOO)_2_, a mixture of metallic barium (0.521 g, 3.8 mmol), tri­phenyl­acetic acid (2.209 g, 7.66 mmol), C_6_H_5_CH_3_ (50 ml) and THF (10 ml) was stirred at 363–373 K for 24 h until all the metal had reacted. The solution, which included a white precipitate, was concentrated to about 20 ml and then hexane (50 ml) was added while stirring, which led to further precipitation. The product was filtered on a Schlenk flask (yield: 2.520 g, 93.26%). Elemental analysis (%) calculated for Ba(Ph_3_CCOO)_2_: C 67.48, H 5.38, Ba 19.29; found: C 67.56, H 5.51, Ba 19.44. Solid ZnCl_2_ (0.273 g, 2.0 mmol) and Ba(Ph_3_CCOO)_2_ (1.426 g, 2.0 mmol) were then added to a solution of CH_3_OCH_2_CH_2_OH (30 ml) and C_6_H_5_CH_3_ (15 ml) and the resulting mixture was stirred under a nitro­gen atmosphere for 24 h. The solution was filtered and then concentrated to about 20 ml. Afterwards 20 ml of hexane was funneled into the reaction solution, leading to the creation of two layers and the mixture was left to crystallize at room temperature. After one week, colorless crystals suitable for the X-ray experiment were obtained (1.289 g, yield: 55.83%). Knowledge of the mol­ecular structure of the final product enables representation of the chemical equation for the reaction as: ZnCl_2_ + Ba((C_6_H_5_)_3_CCOO)_2_ + 4 (CH_3_OCH_2_CH_2_OH) → [BaZnCl_2_[(C_6_H_5_)_3_CCOO]_2_(CH_3_OCH_2_CH_2_OH)_4_]. Elemental analysis: (%) calculated for the complex: C 54.14, H 5.38, Cl 6.3, Zn 5.67, Ba 11.91; found: C 52.94, H 5.67, Zn 5.48, Ba 11.24.

## Refinement details   

Crystal data, data collection and structure refinement details are summarized in Table 3 ▸. All C-bonded H atoms were positioned geometrically and treated as riding atoms: methyl H atoms were constrained to an ideal geometry, with C—H = 0.98 Å and *U*_iso_(H) = 1.5*U*_eq_(C); the remaining H atoms were afixed to C atoms, with C*sp* ^2^—H = 0.95 Å and C*sp* ^3^—H = 0.99 Å, and with *U*_iso_(H) = 1.2*U*_eq_(C). The locations of H atoms of the hydroxyl groups were determined from a difference-Fourier map and finally constrained to ride on their parent atoms, with O—H = 0.84 Å and *U*_iso_(H) = 1.5*U*_eq_(O).

## Supplementary Material

Crystal structure: contains datablock(s) I, publication_text. DOI: 10.1107/S2056989015011226/zs2332sup1.cif

Structure factors: contains datablock(s) I. DOI: 10.1107/S2056989015011226/zs2332Isup2.hkl

CCDC reference: 1405801

Additional supporting information:  crystallographic information; 3D view; checkCIF report

## Figures and Tables

**Table 1:** Selected bond lengths ()

BaO3	2.6925(19)	BaO2*G*	2.985(2)
BaO1	2.7073(19)	BaCl1	3.1118(11)
BaO1*J*	2.7572(19)	ZnO2	1.9682(17)
BaO1*H*	2.783(2)	ZnO4	1.9683(18)
BaO2*J*	2.7908(19)	ZnCl1	2.2595(10)
BaO1*G*	2.799(2)	ZnCl2	2.2653(9)
BaO1*I*	2.810(2)		

**Table 2:** Hydrogen-bond geometry (, )

*D*H*A*	*D*H	H*A*	*D* *A*	*D*H*A*
O1*G*H1*G*O2*I* ^i^	0.84	1.91	2.728(3)	163
O1*I*H1*I*O2*H*	0.84	1.99	2.817(3)	170
C2*I*H2*I*2Cl1^ii^	0.99	2.81	3.660(3)	144
O1*J*H1*J*Cl2^iii^	0.84	2.17	3.012(2)	174

Symmetry codes: (i) 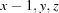; (ii) 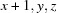; (iii) 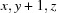.

**Table 3:** Experimental details

Crystal data	
Chemical formula	[BaZn(C_20_H_15_O_2_)_2_Cl_2_(C_3_H_8_O_2_)_4_]
*M*_r_	1152.62
Crystal system, space group	Triclinic, *P* 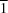
Temperature (K)	100
*a*, *b*, *c* ()	9.706(3), 10.643(3), 25.073(6)
, , ()	89.62(3), 89.26(3), 82.73(3)
*V* (^3^)	2569.0(12)
*Z*	2
Radiation type	Mo *K*
(mm^1^)	1.39
Crystal size (mm)	0.31 0.23 0.21

Data collection	
Diffractometer	Oxford Diffraction KM-4-CCD
Absorption correction	Analytical [*CrysAlis RED* (Oxford Diffraction, 2010 ▸), based on expressions derived by Clark Reid (1995 ▸)]
*T*_min_, *T*_max_	0.687, 0.780
No. of measured, independent and observed [*I* > 2(*I*)] reflections	24098, 12296, 10742
*R*_int_	0.025
(sin /)_max_ (^1^)	0.705

Refinement	
*R*[*F* ^2^ > 2(*F* ^2^)], *wR*(*F* ^2^), *S*	0.035, 0.100, 1.14
No. of reflections	12296
No. of parameters	617
H-atom treatment	H-atom parameters constrained
_max_, _min_ (e ^3^)	0.89, 0.57

Computer programs: *CrysAlis CCD* and *CrysAlis RED* (Oxford Diffraction, 2010 ▸), *SHELXS97* and *SHELXTL* (Sheldrick, 2008 ▸) and *SHELXL2013* (Sheldrick, 2015 ▸).

## supplementary crystallographic information

### Crystal data

**Table:**

[BaZn(C_20_H_15_O_2_)_2_Cl_2_(C_3_H_8_O_2_)_4_]	*Z* = 2
*M**_r_* = 1152.62	*F*(000) = 1180
Triclinic, *P*1	*D*_x_ = 1.490 Mg m^−^^3^
*a* = 9.706 (3) Å	Mo *K*α radiation, λ = 0.71073 Å
*b* = 10.643 (3) Å	Cell parameters from 17769 reflections
*c* = 25.073 (6) Å	θ = 2–31°
α = 89.62 (3)°	µ = 1.39 mm^−^^1^
β = 89.26 (3)°	*T* = 100 K
γ = 82.73 (3)°	Block, colorless
*V* = 2569.0 (12) Å^3^	0.31 × 0.23 × 0.21 mm

### Data collection

**Table:**

Oxford Diffraction KM-4-CCD diffractometer	10742 reflections with *I* > 2σ(*I*)
Radiation source: fine-focus sealed tube	*R*_int_ = 0.025
ω scans	θ_max_ = 30.1°, θ_min_ = 2.8°
Absorption correction: analytical [*CrysAlis RED* (Oxford Diffraction, 2010), based on expressions derived by Clark & Reid (1995)]	*h* = −12→12
*T*_min_ = 0.687, *T*_max_ = 0.780	*k* = −13→13
24098 measured reflections	*l* = −33→35
12296 independent reflections	

### Refinement

**Table:**

Refinement on *F*^2^	Primary atom site location: structure-invariant direct methods
Least-squares matrix: full	Secondary atom site location: difference Fourier map
*R*[*F*^2^ > 2σ(*F*^2^)] = 0.035	Hydrogen site location: inferred from neighbouring sites
*wR*(*F*^2^) = 0.100	H-atom parameters constrained
*S* = 1.14	*w* = 1/[σ^2^(*F*_o_^2^) + (0.064*P*)^2^] where *P* = (*F*_o_^2^ + 2*F*_c_^2^)/3
12296 reflections	(Δ/σ)_max_ = 0.001
617 parameters	Δρ_max_ = 0.89 e Å^−^^3^
0 restraints	Δρ_min_ = −0.57 e Å^−^^3^

### Special details

**Table:**

Experimental. The O-bonded H atoms were found from a difference-Fourier map. These H atoms were included in the refinement with constraint:;finally with instruction Afix 3.Absorption correction: CrysAlis RED (Oxford Diffraction, 2010), employing an analytical numeric absorption correction using a multifaceted crystal model based on expressions derived by R.C. Clark & J.S. Reid (Clark & Reid, 1995).
Geometry. All e.s.d.'s (except the e.s.d. in the dihedral angle between two l.s. planes) are estimated using the full covariance matrix. The cell e.s.d.'s are taken into account individually in the estimation of e.s.d.'s in distances, angles and torsion angles; correlations between e.s.d.'s in cell parameters are only used when they are defined by crystal symmetry. An approximate (isotropic) treatment of cell e.s.d.'s is used for estimating e.s.d.'s involving l.s. planes.
Refinement. Refinement of *F*^2^ against ALL reflections. The weighted *R*-factor *wR* and goodness of fit *S* are based on *F*^2^, conventional *R*-factors *R* are based on *F*, with *F* set to zero for negative *F*^2^. The threshold expression of *F*^2^ > σ(*F*^2^) is used only for calculating *R*-factors(gt) *etc*. and is not relevant to the choice of reflections for refinement. *R*-factors based on *F*^2^ are statistically about twice as large as those based on *F*, and *R*- factors based on ALL data will be even larger.

### Fractional atomic coordinates and isotropic or equivalent isotropic displacement parameters (Å^2^)

**Table:**

	*x*	*y*	*z*	*U*_iso_*/*U*_eq_	
Ba	0.13461 (2)	0.39066 (2)	0.25149 (2)	0.01587 (5)	
Zn	0.09859 (3)	0.02666 (2)	0.25185 (2)	0.01406 (7)	
Cl1	−0.07279 (6)	0.19070 (6)	0.25860 (3)	0.02295 (13)	
Cl2	−0.00737 (6)	−0.14768 (6)	0.23751 (2)	0.02093 (12)	
O1	0.24715 (19)	0.19812 (16)	0.31457 (7)	0.0202 (4)	
O2	0.20140 (18)	−0.00059 (16)	0.31895 (6)	0.0178 (3)	
C1	0.2527 (2)	0.0946 (2)	0.33730 (9)	0.0147 (4)	
C2	0.3316 (2)	0.0704 (2)	0.39131 (9)	0.0131 (4)	
C1A	0.3078 (2)	0.1863 (2)	0.42871 (9)	0.0141 (4)	
C2A	0.3301 (3)	0.3070 (2)	0.41065 (9)	0.0196 (5)	
H2A	0.3540	0.3190	0.3743	0.024*	
C3A	0.3179 (3)	0.4090 (2)	0.44516 (10)	0.0238 (5)	
H3A	0.3340	0.4900	0.4321	0.029*	
C4A	0.2824 (3)	0.3948 (2)	0.49867 (10)	0.0247 (5)	
H4A	0.2729	0.4654	0.5220	0.030*	
C5A	0.2613 (3)	0.2763 (2)	0.51715 (10)	0.0222 (5)	
H5A	0.2372	0.2648	0.5535	0.027*	
C6A	0.2752 (3)	0.1736 (2)	0.48271 (9)	0.0180 (5)	
H6A	0.2621	0.0923	0.4963	0.022*	
C1B	0.2833 (2)	−0.0447 (2)	0.41952 (9)	0.0148 (4)	
C2B	0.3746 (3)	−0.1460 (2)	0.43841 (9)	0.0175 (5)	
H2B	0.4712	−0.1488	0.4311	0.021*	
C3B	0.3268 (3)	−0.2436 (2)	0.46791 (10)	0.0217 (5)	
H3B	0.3907	−0.3120	0.4805	0.026*	
C4B	0.1867 (3)	−0.2410 (2)	0.47891 (10)	0.0233 (5)	
H4B	0.1543	−0.3071	0.4993	0.028*	
C5B	0.0933 (3)	−0.1414 (2)	0.46006 (10)	0.0208 (5)	
H5B	−0.0033	−0.1393	0.4673	0.025*	
C6B	0.1418 (3)	−0.0450 (2)	0.43054 (9)	0.0177 (5)	
H6B	0.0773	0.0224	0.4175	0.021*	
C1C	0.4882 (2)	0.0448 (2)	0.37655 (9)	0.0144 (4)	
C2C	0.5884 (3)	0.0737 (2)	0.41205 (9)	0.0187 (5)	
H2C	0.5599	0.1150	0.4446	0.022*	
C3C	0.7289 (3)	0.0433 (2)	0.40100 (10)	0.0216 (5)	
H3C	0.7955	0.0634	0.4259	0.026*	
C4C	0.7723 (3)	−0.0164 (3)	0.35348 (10)	0.0233 (5)	
H4C	0.8684	−0.0380	0.3458	0.028*	
C5C	0.6735 (3)	−0.0441 (3)	0.31737 (10)	0.0256 (6)	
H5C	0.7023	−0.0844	0.2847	0.031*	
C6C	0.5329 (3)	−0.0136 (2)	0.32859 (10)	0.0203 (5)	
H6C	0.4664	−0.0327	0.3034	0.024*	
O3	0.2094 (2)	0.22740 (16)	0.17158 (7)	0.0244 (4)	
O4	0.22946 (18)	0.02259 (16)	0.19108 (6)	0.0195 (4)	
C3	0.2431 (3)	0.1152 (2)	0.16032 (9)	0.0167 (5)	
C4	0.3131 (2)	0.0801 (2)	0.10490 (9)	0.0164 (4)	
C1D	0.3201 (3)	0.2003 (2)	0.07098 (9)	0.0189 (5)	
C2D	0.4423 (3)	0.2304 (3)	0.04765 (10)	0.0245 (5)	
H2D	0.5275	0.1794	0.0551	0.029*	
C3D	0.4417 (4)	0.3342 (3)	0.01355 (11)	0.0335 (7)	
H3D	0.5264	0.3524	−0.0024	0.040*	
C4D	0.3199 (4)	0.4111 (3)	0.00251 (12)	0.0355 (7)	
H4D	0.3198	0.4817	−0.0208	0.043*	
C5D	0.1979 (4)	0.3830 (3)	0.02615 (12)	0.0356 (7)	
H5D	0.1133	0.4356	0.0194	0.043*	
C6D	0.1978 (3)	0.2785 (3)	0.05973 (11)	0.0253 (5)	
H6D	0.1128	0.2601	0.0753	0.030*	
C1E	0.4604 (2)	0.0178 (2)	0.11792 (9)	0.0175 (5)	
C2E	0.5232 (3)	−0.0894 (2)	0.09188 (9)	0.0201 (5)	
H2E	0.4725	−0.1297	0.0663	0.024*	
C3E	0.6595 (3)	−0.1387 (3)	0.10280 (10)	0.0248 (5)	
H3E	0.7010	−0.2120	0.0845	0.030*	
C4E	0.7351 (3)	−0.0822 (3)	0.13997 (11)	0.0281 (6)	
H4E	0.8285	−0.1159	0.1470	0.034*	
C5E	0.6736 (3)	0.0245 (3)	0.16711 (11)	0.0283 (6)	
H5E	0.7244	0.0634	0.1931	0.034*	
C6E	0.5379 (3)	0.0737 (3)	0.15608 (10)	0.0248 (5)	
H6E	0.4964	0.1466	0.1747	0.030*	
C1F	0.2325 (2)	−0.0103 (2)	0.07292 (9)	0.0167 (5)	
C2F	0.2503 (3)	−0.0153 (3)	0.01745 (10)	0.0249 (5)	
H2F	0.3067	0.0402	0.0005	0.030*	
C3F	0.1875 (3)	−0.0990 (3)	−0.01334 (10)	0.0277 (6)	
H3F	0.2015	−0.1000	−0.0509	0.033*	
C4F	0.1055 (3)	−0.1806 (3)	0.00986 (11)	0.0296 (6)	
H4F	0.0646	−0.2398	−0.0110	0.036*	
C5F	0.0841 (3)	−0.1744 (3)	0.06440 (12)	0.0351 (7)	
H5F	0.0253	−0.2285	0.0809	0.042*	
C6F	0.1463 (3)	−0.0909 (3)	0.09569 (11)	0.0279 (6)	
H6F	0.1298	−0.0890	0.1331	0.034*	
O1G	−0.12372 (19)	0.54119 (18)	0.25180 (8)	0.0257 (4)	
H1G	−0.1790	0.5348	0.2774	0.039*	
C1G	−0.1947 (3)	0.5900 (3)	0.20479 (11)	0.0276 (6)	
H1G1	−0.2228	0.6823	0.2081	0.033*	
H1G2	−0.2792	0.5484	0.1996	0.033*	
C2G	−0.0963 (3)	0.5630 (3)	0.15848 (12)	0.0299 (6)	
H2G1	−0.1454	0.5896	0.1251	0.036*	
H2G2	−0.0186	0.6142	0.1621	0.036*	
O2G	−0.0411 (2)	0.43219 (19)	0.15458 (8)	0.0293 (4)	
C3G	−0.1399 (4)	0.3533 (3)	0.13765 (13)	0.0390 (7)	
H3G1	−0.2184	0.3603	0.1629	0.058*	
H3G2	−0.0962	0.2652	0.1363	0.058*	
H3G3	−0.1732	0.3802	0.1021	0.058*	
O1H	0.2695 (2)	0.4717 (2)	0.16137 (8)	0.0352 (5)	
H1H	0.2743	0.4048	0.1435	0.053*	
C1H	0.3414 (4)	0.5586 (3)	0.13328 (13)	0.0425 (8)	
H1H1	0.2929	0.5839	0.0996	0.051*	
H1H2	0.4365	0.5188	0.1243	0.051*	
C2H	0.3480 (4)	0.6720 (3)	0.16707 (13)	0.0366 (7)	
H2H1	0.3783	0.7415	0.1452	0.044*	
H2H2	0.2549	0.7013	0.1823	0.044*	
O2H	0.4452 (2)	0.63873 (17)	0.20935 (8)	0.0255 (4)	
C3H	0.4607 (3)	0.7504 (3)	0.23808 (13)	0.0328 (6)	
H3H1	0.4842	0.8161	0.2132	0.049*	
H3H2	0.5352	0.7320	0.2641	0.049*	
H3H3	0.3735	0.7801	0.2568	0.049*	
O1I	0.40709 (19)	0.43145 (18)	0.27599 (8)	0.0280 (4)	
H1I	0.4267	0.4943	0.2583	0.042*	
C1I	0.5361 (3)	0.3481 (3)	0.27592 (12)	0.0294 (6)	
H1I1	0.5877	0.3600	0.2423	0.035*	
H1I2	0.5162	0.2591	0.2773	0.035*	
C2I	0.6245 (3)	0.3723 (3)	0.32224 (12)	0.0327 (6)	
H2I1	0.5721	0.3626	0.3558	0.039*	
H2I2	0.7083	0.3088	0.3224	0.039*	
O2I	0.6661 (2)	0.4974 (2)	0.31999 (8)	0.0334 (5)	
C3I	0.5953 (4)	0.5833 (4)	0.35674 (13)	0.0435 (8)	
H3I1	0.4962	0.5968	0.3482	0.065*	
H3I2	0.6334	0.6642	0.3546	0.065*	
H3I3	0.6070	0.5485	0.3929	0.065*	
O1J	0.1679 (2)	0.62932 (17)	0.28717 (7)	0.0235 (4)	
H1J	0.1135	0.6889	0.2740	0.035*	
C1J	0.1615 (3)	0.6484 (3)	0.34373 (11)	0.0279 (6)	
H1J1	0.1530	0.7403	0.3513	0.033*	
H1J2	0.2487	0.6079	0.3599	0.033*	
C2J	0.0403 (3)	0.5938 (3)	0.36864 (11)	0.0273 (6)	
H2J1	0.0414	0.6034	0.4079	0.033*	
H2J2	−0.0479	0.6391	0.3552	0.033*	
O2J	0.0511 (2)	0.46206 (17)	0.35502 (7)	0.0238 (4)	
C3J	−0.0263 (3)	0.3905 (3)	0.38985 (11)	0.0300 (6)	
H3J1	0.0141	0.3877	0.4255	0.045*	
H3J2	−0.0234	0.3041	0.3762	0.045*	
H3J3	−0.1229	0.4304	0.3918	0.045*	

### Atomic displacement parameters (Å^2^)

**Table:**

	*U*^11^	*U*^22^	*U*^33^	*U*^12^	*U*^13^	*U*^23^
Ba	0.01883 (8)	0.01347 (8)	0.01522 (8)	−0.00166 (5)	−0.00076 (5)	−0.00073 (5)
Zn	0.01664 (14)	0.01436 (14)	0.01128 (12)	−0.00221 (10)	−0.00157 (10)	−0.00034 (9)
Cl1	0.0179 (3)	0.0180 (3)	0.0328 (3)	−0.0018 (2)	−0.0001 (2)	−0.0005 (2)
Cl2	0.0215 (3)	0.0179 (3)	0.0242 (3)	−0.0053 (2)	−0.0049 (2)	−0.0010 (2)
O1	0.0276 (9)	0.0181 (8)	0.0157 (8)	−0.0051 (7)	−0.0059 (7)	0.0044 (6)
O2	0.0229 (9)	0.0175 (8)	0.0136 (7)	−0.0042 (7)	−0.0039 (7)	−0.0004 (6)
C1	0.0158 (11)	0.0151 (11)	0.0127 (10)	0.0005 (8)	0.0001 (8)	−0.0002 (8)
C2	0.0152 (11)	0.0121 (10)	0.0123 (10)	−0.0030 (8)	−0.0005 (8)	0.0006 (8)
C1A	0.0126 (10)	0.0153 (11)	0.0144 (10)	−0.0010 (8)	−0.0017 (8)	−0.0021 (8)
C2A	0.0262 (13)	0.0184 (12)	0.0150 (10)	−0.0057 (10)	−0.0037 (9)	0.0002 (9)
C3A	0.0340 (15)	0.0139 (12)	0.0234 (12)	−0.0017 (10)	−0.0070 (11)	−0.0015 (9)
C4A	0.0302 (14)	0.0203 (13)	0.0227 (12)	0.0013 (10)	−0.0057 (11)	−0.0065 (10)
C5A	0.0237 (13)	0.0279 (13)	0.0149 (11)	−0.0028 (10)	−0.0008 (9)	−0.0042 (9)
C6A	0.0220 (12)	0.0181 (11)	0.0143 (10)	−0.0039 (9)	0.0004 (9)	−0.0018 (9)
C1B	0.0211 (12)	0.0128 (10)	0.0110 (9)	−0.0042 (9)	−0.0008 (8)	−0.0018 (8)
C2B	0.0221 (12)	0.0152 (11)	0.0152 (10)	−0.0018 (9)	−0.0033 (9)	−0.0012 (8)
C3B	0.0323 (14)	0.0147 (11)	0.0184 (11)	−0.0040 (10)	−0.0043 (10)	0.0012 (9)
C4B	0.0373 (15)	0.0184 (12)	0.0168 (11)	−0.0131 (11)	0.0029 (10)	0.0005 (9)
C5B	0.0230 (12)	0.0228 (12)	0.0183 (11)	−0.0097 (10)	0.0045 (9)	−0.0038 (9)
C6B	0.0196 (12)	0.0166 (11)	0.0162 (10)	0.0000 (9)	0.0012 (9)	−0.0021 (9)
C1C	0.0163 (11)	0.0136 (10)	0.0132 (10)	−0.0013 (8)	0.0016 (8)	0.0017 (8)
C2C	0.0203 (12)	0.0227 (12)	0.0132 (10)	−0.0032 (10)	0.0005 (9)	−0.0014 (9)
C3C	0.0178 (12)	0.0271 (13)	0.0203 (11)	−0.0046 (10)	−0.0033 (9)	0.0023 (10)
C4C	0.0151 (12)	0.0284 (14)	0.0255 (13)	−0.0003 (10)	0.0047 (10)	0.0026 (10)
C5C	0.0244 (13)	0.0321 (15)	0.0200 (12)	−0.0027 (11)	0.0071 (10)	−0.0068 (10)
C6C	0.0188 (12)	0.0251 (13)	0.0176 (11)	−0.0045 (10)	0.0009 (9)	−0.0061 (9)
O3	0.0364 (11)	0.0157 (9)	0.0210 (9)	−0.0033 (8)	0.0081 (8)	−0.0044 (7)
O4	0.0247 (9)	0.0196 (9)	0.0141 (8)	−0.0032 (7)	0.0025 (7)	0.0011 (6)
C3	0.0184 (11)	0.0199 (12)	0.0123 (10)	−0.0040 (9)	0.0000 (9)	0.0000 (8)
C4	0.0180 (11)	0.0159 (11)	0.0154 (10)	−0.0028 (9)	−0.0002 (9)	0.0007 (8)
C1D	0.0248 (13)	0.0197 (12)	0.0131 (10)	−0.0059 (10)	−0.0019 (9)	−0.0012 (9)
C2D	0.0304 (14)	0.0247 (13)	0.0199 (12)	−0.0092 (11)	0.0040 (10)	−0.0016 (10)
C3D	0.0497 (19)	0.0309 (15)	0.0241 (13)	−0.0219 (13)	0.0011 (13)	0.0016 (11)
C4D	0.061 (2)	0.0227 (14)	0.0263 (14)	−0.0173 (14)	−0.0104 (14)	0.0082 (11)
C5D	0.0477 (19)	0.0240 (14)	0.0355 (16)	−0.0046 (13)	−0.0183 (14)	0.0061 (12)
C6D	0.0279 (14)	0.0238 (13)	0.0243 (12)	−0.0033 (11)	−0.0052 (11)	0.0025 (10)
C1E	0.0178 (11)	0.0209 (12)	0.0139 (10)	−0.0038 (9)	0.0010 (9)	0.0019 (9)
C2E	0.0226 (13)	0.0230 (12)	0.0147 (10)	−0.0039 (10)	0.0004 (9)	0.0022 (9)
C3E	0.0239 (13)	0.0275 (14)	0.0221 (12)	0.0002 (11)	−0.0008 (10)	0.0024 (10)
C4E	0.0201 (13)	0.0392 (16)	0.0241 (13)	−0.0008 (11)	−0.0027 (10)	0.0097 (11)
C5E	0.0267 (14)	0.0383 (16)	0.0215 (12)	−0.0105 (12)	−0.0067 (11)	−0.0003 (11)
C6E	0.0246 (13)	0.0297 (14)	0.0213 (12)	−0.0077 (11)	−0.0010 (10)	−0.0029 (10)
C1F	0.0172 (11)	0.0178 (11)	0.0149 (10)	−0.0014 (9)	−0.0021 (9)	−0.0023 (8)
C2F	0.0316 (14)	0.0273 (14)	0.0173 (11)	−0.0092 (11)	0.0010 (10)	−0.0006 (10)
C3F	0.0324 (15)	0.0331 (15)	0.0179 (12)	−0.0053 (12)	−0.0009 (11)	−0.0021 (10)
C4F	0.0280 (14)	0.0361 (16)	0.0270 (13)	−0.0120 (12)	−0.0045 (11)	−0.0082 (11)
C5F	0.0407 (17)	0.0426 (18)	0.0274 (14)	−0.0269 (14)	0.0036 (13)	−0.0037 (12)
C6F	0.0339 (15)	0.0336 (15)	0.0189 (12)	−0.0143 (12)	0.0023 (11)	−0.0029 (10)
O1G	0.0219 (9)	0.0263 (10)	0.0283 (10)	−0.0015 (7)	−0.0003 (8)	0.0034 (8)
C1G	0.0227 (13)	0.0237 (13)	0.0357 (15)	0.0000 (11)	−0.0055 (11)	0.0048 (11)
C2G	0.0265 (14)	0.0283 (14)	0.0341 (15)	−0.0010 (11)	−0.0035 (12)	0.0108 (11)
O2G	0.0281 (10)	0.0294 (10)	0.0291 (10)	0.0013 (8)	−0.0033 (8)	0.0011 (8)
C3G	0.0416 (18)	0.0429 (18)	0.0327 (16)	−0.0052 (14)	−0.0105 (14)	−0.0042 (13)
O1H	0.0540 (14)	0.0334 (11)	0.0229 (10)	−0.0244 (10)	0.0042 (9)	−0.0042 (8)
C1H	0.056 (2)	0.049 (2)	0.0284 (15)	−0.0296 (17)	0.0075 (14)	−0.0014 (13)
C2H	0.0457 (19)	0.0329 (16)	0.0339 (16)	−0.0152 (14)	−0.0050 (14)	0.0050 (12)
O2H	0.0266 (10)	0.0213 (9)	0.0286 (10)	−0.0032 (7)	−0.0051 (8)	−0.0014 (7)
C3H	0.0376 (17)	0.0208 (14)	0.0404 (16)	−0.0056 (12)	0.0005 (13)	−0.0063 (12)
O1I	0.0222 (10)	0.0286 (10)	0.0338 (10)	−0.0052 (8)	−0.0057 (8)	0.0094 (8)
C1I	0.0243 (14)	0.0274 (14)	0.0356 (15)	−0.0003 (11)	0.0037 (11)	−0.0007 (11)
C2I	0.0244 (14)	0.0381 (17)	0.0343 (15)	0.0004 (12)	−0.0005 (12)	0.0103 (13)
O2I	0.0245 (10)	0.0441 (13)	0.0321 (11)	−0.0066 (9)	0.0032 (8)	−0.0031 (9)
C3I	0.046 (2)	0.055 (2)	0.0287 (15)	−0.0026 (16)	−0.0039 (14)	−0.0069 (14)
O1J	0.0294 (10)	0.0184 (9)	0.0221 (9)	0.0001 (7)	−0.0021 (8)	−0.0024 (7)
C1J	0.0347 (15)	0.0243 (14)	0.0251 (13)	−0.0052 (11)	−0.0037 (11)	−0.0033 (10)
C2J	0.0327 (15)	0.0253 (14)	0.0231 (12)	−0.0003 (11)	0.0020 (11)	−0.0073 (10)
O2J	0.0286 (10)	0.0231 (9)	0.0194 (8)	−0.0025 (8)	0.0035 (7)	0.0005 (7)
C3J	0.0266 (14)	0.0391 (16)	0.0253 (13)	−0.0084 (12)	0.0014 (11)	0.0032 (11)

### Geometric parameters (Å, º)

**Table:**

Ba—O3	2.6925 (19)	C1E—C2E	1.387 (3)
Ba—O1	2.7073 (19)	C1E—C6E	1.405 (3)
Ba—O1J	2.7572 (19)	C2E—C3E	1.389 (4)
Ba—O1H	2.783 (2)	C2E—H2E	0.9500
Ba—O2J	2.7908 (19)	C3E—C4E	1.381 (4)
Ba—O1G	2.799 (2)	C3E—H3E	0.9500
Ba—O1I	2.810 (2)	C4E—C5E	1.392 (4)
Ba—O2G	2.985 (2)	C4E—H4E	0.9500
Ba—Cl1	3.1118 (11)	C5E—C6E	1.385 (4)
Ba—Zn	3.9335 (11)	C5E—H5E	0.9500
Zn—O2	1.9682 (17)	C6E—H6E	0.9500
Zn—O4	1.9683 (18)	C1F—C6F	1.388 (4)
Zn—Cl1	2.2595 (10)	C1F—C2F	1.400 (3)
Zn—Cl2	2.2653 (9)	C2F—C3F	1.386 (4)
O1—C1	1.233 (3)	C2F—H2F	0.9500
O2—C1	1.275 (3)	C3F—C4F	1.372 (4)
C1—C2	1.568 (3)	C3F—H3F	0.9500
C2—C1B	1.531 (3)	C4F—C5F	1.381 (4)
C2—C1A	1.547 (3)	C4F—H4F	0.9500
C2—C1C	1.550 (3)	C5F—C6F	1.388 (4)
C1A—C6A	1.396 (3)	C5F—H5F	0.9500
C1A—C2A	1.401 (3)	C6F—H6F	0.9500
C2A—C3A	1.385 (3)	O1G—C1G	1.434 (3)
C2A—H2A	0.9500	O1G—H1G	0.8397
C3A—C4A	1.393 (4)	C1G—C2G	1.500 (4)
C3A—H3A	0.9500	C1G—H1G1	0.9900
C4A—C5A	1.380 (4)	C1G—H1G2	0.9900
C4A—H4A	0.9500	C2G—O2G	1.431 (3)
C5A—C6A	1.390 (3)	C2G—H2G1	0.9900
C5A—H5A	0.9500	C2G—H2G2	0.9900
C6A—H6A	0.9500	O2G—C3G	1.422 (4)
C1B—C2B	1.391 (3)	C3G—H3G1	0.9800
C1B—C6B	1.397 (3)	C3G—H3G2	0.9800
C2B—C3B	1.394 (3)	C3G—H3G3	0.9800
C2B—H2B	0.9500	O1H—C1H	1.407 (4)
C3B—C4B	1.381 (4)	O1H—H1H	0.8402
C3B—H3B	0.9500	C1H—C2H	1.487 (4)
C4B—C5B	1.389 (4)	C1H—H1H1	0.9900
C4B—H4B	0.9500	C1H—H1H2	0.9900
C5B—C6B	1.388 (3)	C2H—O2H	1.440 (4)
C5B—H5B	0.9500	C2H—H2H1	0.9900
C6B—H6B	0.9500	C2H—H2H2	0.9900
C1C—C2C	1.390 (3)	O2H—C3H	1.419 (3)
C1C—C6C	1.396 (3)	C3H—H3H1	0.9800
C2C—C3C	1.386 (3)	C3H—H3H2	0.9800
C2C—H2C	0.9500	C3H—H3H3	0.9800
C3C—C4C	1.390 (4)	O1I—C1I	1.440 (3)
C3C—H3C	0.9500	O1I—H1I	0.8399
C4C—C5C	1.387 (4)	C1I—C2I	1.496 (4)
C4C—H4C	0.9500	C1I—H1I1	0.9900
C5C—C6C	1.388 (4)	C1I—H1I2	0.9900
C5C—H5C	0.9500	C2I—O2I	1.439 (4)
C6C—H6C	0.9500	C2I—H2I1	0.9900
O3—C3	1.232 (3)	C2I—H2I2	0.9900
O4—C3	1.266 (3)	O2I—C3I	1.412 (4)
C3—C4	1.565 (3)	C3I—H3I1	0.9800
C4—C1E	1.536 (3)	C3I—H3I2	0.9800
C4—C1D	1.540 (3)	C3I—H3I3	0.9800
C4—C1F	1.549 (3)	O1J—C1J	1.433 (3)
C1D—C2D	1.388 (4)	O1J—H1J	0.8403
C1D—C6D	1.391 (4)	C1J—C2J	1.503 (4)
C2D—C3D	1.392 (4)	C1J—H1J1	0.9900
C2D—H2D	0.9500	C1J—H1J2	0.9900
C3D—C4D	1.380 (5)	C2J—O2J	1.436 (3)
C3D—H3D	0.9500	C2J—H2J1	0.9900
C4D—C5D	1.383 (5)	C2J—H2J2	0.9900
C4D—H4D	0.9500	O2J—C3J	1.424 (3)
C5D—C6D	1.390 (4)	C3J—H3J1	0.9800
C5D—H5D	0.9500	C3J—H3J2	0.9800
C6D—H6D	0.9500	C3J—H3J3	0.9800
			
O3—Ba—O1	84.08 (6)	C4D—C3D—H3D	119.5
O3—Ba—O1J	142.04 (6)	C2D—C3D—H3D	119.5
O1—Ba—O1J	114.85 (6)	C3D—C4D—C5D	118.6 (3)
O3—Ba—O1H	60.16 (6)	C3D—C4D—H4D	120.7
O1—Ba—O1H	122.95 (7)	C5D—C4D—H4D	120.7
O1J—Ba—O1H	82.35 (6)	C4D—C5D—C6D	120.7 (3)
O3—Ba—O2J	155.84 (6)	C4D—C5D—H5D	119.7
O1—Ba—O2J	74.87 (6)	C6D—C5D—H5D	119.7
O1J—Ba—O2J	60.31 (6)	C5D—C6D—C1D	121.2 (3)
O1H—Ba—O2J	142.49 (6)	C5D—C6D—H6D	119.4
O3—Ba—O1G	120.96 (6)	C1D—C6D—H6D	119.4
O1—Ba—O1G	133.04 (6)	C2E—C1E—C6E	118.1 (2)
O1J—Ba—O1G	71.05 (6)	C2E—C1E—C4	122.6 (2)
O1H—Ba—O1G	103.93 (7)	C6E—C1E—C4	119.3 (2)
O2J—Ba—O1G	68.94 (6)	C1E—C2E—C3E	120.8 (2)
O3—Ba—O1I	95.18 (7)	C1E—C2E—H2E	119.6
O1—Ba—O1I	71.58 (6)	C3E—C2E—H2E	119.6
O1J—Ba—O1I	63.80 (6)	C4E—C3E—C2E	120.7 (3)
O1H—Ba—O1I	69.50 (7)	C4E—C3E—H3E	119.7
O2J—Ba—O1I	89.38 (6)	C2E—C3E—H3E	119.7
O1G—Ba—O1I	134.83 (6)	C3E—C4E—C5E	119.6 (3)
O3—Ba—O2G	65.59 (6)	C3E—C4E—H4E	120.2
O1—Ba—O2G	139.45 (6)	C5E—C4E—H4E	120.2
O1J—Ba—O2G	105.51 (6)	C6E—C5E—C4E	119.7 (3)
O1H—Ba—O2G	64.98 (6)	C6E—C5E—H5E	120.2
O2J—Ba—O2G	125.48 (6)	C4E—C5E—H5E	120.2
O1G—Ba—O2G	57.15 (6)	C5E—C6E—C1E	121.2 (3)
O1I—Ba—O2G	134.31 (6)	C5E—C6E—H6E	119.4
O3—Ba—Cl1	74.73 (5)	C1E—C6E—H6E	119.4
O1—Ba—Cl1	71.96 (5)	C6F—C1F—C2F	117.0 (2)
O1J—Ba—Cl1	140.95 (4)	C6F—C1F—C4	124.4 (2)
O1H—Ba—Cl1	128.16 (5)	C2F—C1F—C4	118.6 (2)
O2J—Ba—Cl1	87.39 (5)	C3F—C2F—C1F	121.7 (2)
O1G—Ba—Cl1	77.39 (5)	C3F—C2F—H2F	119.2
O1I—Ba—Cl1	142.95 (4)	C1F—C2F—H2F	119.2
O2G—Ba—Cl1	74.38 (5)	C4F—C3F—C2F	120.7 (2)
O3—Ba—Zn	53.90 (4)	C4F—C3F—H3F	119.6
O1—Ba—Zn	48.34 (4)	C2F—C3F—H3F	119.6
O1J—Ba—Zn	160.81 (4)	C3F—C4F—C5F	118.2 (3)
O1H—Ba—Zn	113.88 (5)	C3F—C4F—H4F	120.9
O2J—Ba—Zn	102.37 (5)	C5F—C4F—H4F	120.9
O1G—Ba—Zn	112.26 (5)	C4F—C5F—C6F	121.6 (3)
O1I—Ba—Zn	110.85 (5)	C4F—C5F—H5F	119.2
O2G—Ba—Zn	91.21 (5)	C6F—C5F—H5F	119.2
Cl1—Ba—Zn	35.01 (2)	C1F—C6F—C5F	120.7 (2)
O2—Zn—O4	110.00 (8)	C1F—C6F—H6F	119.6
O2—Zn—Cl1	111.30 (6)	C5F—C6F—H6F	119.6
O4—Zn—Cl1	118.74 (6)	C1G—O1G—Ba	124.47 (16)
O2—Zn—Cl2	107.44 (6)	C1G—O1G—H1G	111.8
O4—Zn—Cl2	102.14 (6)	Ba—O1G—H1G	119.0
Cl1—Zn—Cl2	106.19 (3)	O1G—C1G—C2G	107.4 (2)
O2—Zn—Ba	91.95 (6)	O1G—C1G—H1G1	110.2
O4—Zn—Ba	83.59 (6)	C2G—C1G—H1G1	110.2
Cl1—Zn—Ba	52.20 (3)	O1G—C1G—H1G2	110.2
Cl2—Zn—Ba	156.02 (2)	C2G—C1G—H1G2	110.2
Zn—Cl1—Ba	92.79 (3)	H1G1—C1G—H1G2	108.5
C1—O1—Ba	155.72 (16)	O2G—C2G—C1G	113.0 (2)
C1—O2—Zn	116.35 (15)	O2G—C2G—H2G1	109.0
O1—C1—O2	124.3 (2)	C1G—C2G—H2G1	109.0
O1—C1—C2	120.6 (2)	O2G—C2G—H2G2	109.0
O2—C1—C2	115.06 (19)	C1G—C2G—H2G2	109.0
C1B—C2—C1A	109.37 (18)	H2G1—C2G—H2G2	107.8
C1B—C2—C1C	110.82 (18)	C3G—O2G—C2G	113.5 (2)
C1A—C2—C1C	108.82 (18)	C3G—O2G—Ba	125.66 (17)
C1B—C2—C1	109.40 (18)	C2G—O2G—Ba	102.89 (15)
C1A—C2—C1	112.19 (18)	O2G—C3G—H3G1	109.5
C1C—C2—C1	106.20 (17)	O2G—C3G—H3G2	109.5
C6A—C1A—C2A	117.2 (2)	H3G1—C3G—H3G2	109.5
C6A—C1A—C2	121.6 (2)	O2G—C3G—H3G3	109.5
C2A—C1A—C2	121.0 (2)	H3G1—C3G—H3G3	109.5
C3A—C2A—C1A	120.8 (2)	H3G2—C3G—H3G3	109.5
C3A—C2A—H2A	119.6	C1H—O1H—Ba	152.75 (18)
C1A—C2A—H2A	119.6	C1H—O1H—H1H	108.3
C2A—C3A—C4A	121.1 (2)	Ba—O1H—H1H	98.5
C2A—C3A—H3A	119.5	O1H—C1H—C2H	109.1 (3)
C4A—C3A—H3A	119.5	O1H—C1H—H1H1	109.9
C5A—C4A—C3A	118.8 (2)	C2H—C1H—H1H1	109.9
C5A—C4A—H4A	120.6	O1H—C1H—H1H2	109.9
C3A—C4A—H4A	120.6	C2H—C1H—H1H2	109.9
C4A—C5A—C6A	120.2 (2)	H1H1—C1H—H1H2	108.3
C4A—C5A—H5A	119.9	O2H—C2H—C1H	108.8 (3)
C6A—C5A—H5A	119.9	O2H—C2H—H2H1	109.9
C5A—C6A—C1A	121.9 (2)	C1H—C2H—H2H1	109.9
C5A—C6A—H6A	119.0	O2H—C2H—H2H2	109.9
C1A—C6A—H6A	119.0	C1H—C2H—H2H2	109.9
C2B—C1B—C6B	117.7 (2)	H2H1—C2H—H2H2	108.3
C2B—C1B—C2	123.1 (2)	C3H—O2H—C2H	108.2 (2)
C6B—C1B—C2	119.1 (2)	O2H—C3H—H3H1	109.5
C1B—C2B—C3B	121.2 (2)	O2H—C3H—H3H2	109.5
C1B—C2B—H2B	119.4	H3H1—C3H—H3H2	109.5
C3B—C2B—H2B	119.4	O2H—C3H—H3H3	109.5
C4B—C3B—C2B	120.1 (2)	H3H1—C3H—H3H3	109.5
C4B—C3B—H3B	119.9	H3H2—C3H—H3H3	109.5
C2B—C3B—H3B	119.9	C1I—O1I—Ba	131.91 (16)
C3B—C4B—C5B	119.8 (2)	C1I—O1I—H1I	103.1
C3B—C4B—H4B	120.1	Ba—O1I—H1I	108.4
C5B—C4B—H4B	120.1	O1I—C1I—C2I	111.8 (2)
C6B—C5B—C4B	119.6 (2)	O1I—C1I—H1I1	109.3
C6B—C5B—H5B	120.2	C2I—C1I—H1I1	109.3
C4B—C5B—H5B	120.2	O1I—C1I—H1I2	109.3
C5B—C6B—C1B	121.6 (2)	C2I—C1I—H1I2	109.3
C5B—C6B—H6B	119.2	H1I1—C1I—H1I2	107.9
C1B—C6B—H6B	119.2	O2I—C2I—C1I	111.8 (2)
C2C—C1C—C6C	118.1 (2)	O2I—C2I—H2I1	109.3
C2C—C1C—C2	120.7 (2)	C1I—C2I—H2I1	109.3
C6C—C1C—C2	121.0 (2)	O2I—C2I—H2I2	109.3
C3C—C2C—C1C	121.4 (2)	C1I—C2I—H2I2	109.3
C3C—C2C—H2C	119.3	H2I1—C2I—H2I2	107.9
C1C—C2C—H2C	119.3	C3I—O2I—C2I	114.1 (3)
C2C—C3C—C4C	120.0 (2)	O2I—C3I—H3I1	109.5
C2C—C3C—H3C	120.0	O2I—C3I—H3I2	109.5
C4C—C3C—H3C	120.0	H3I1—C3I—H3I2	109.5
C5C—C4C—C3C	119.2 (2)	O2I—C3I—H3I3	109.5
C5C—C4C—H4C	120.4	H3I1—C3I—H3I3	109.5
C3C—C4C—H4C	120.4	H3I2—C3I—H3I3	109.5
C4C—C5C—C6C	120.6 (2)	C1J—O1J—Ba	116.81 (15)
C4C—C5C—H5C	119.7	C1J—O1J—H1J	105.9
C6C—C5C—H5C	119.7	Ba—O1J—H1J	115.3
C5C—C6C—C1C	120.7 (2)	O1J—C1J—C2J	111.5 (2)
C5C—C6C—H6C	119.7	O1J—C1J—H1J1	109.3
C1C—C6C—H6C	119.7	C2J—C1J—H1J1	109.3
C3—O3—Ba	144.65 (16)	O1J—C1J—H1J2	109.3
C3—O4—Zn	125.09 (16)	C2J—C1J—H1J2	109.3
O3—C3—O4	125.0 (2)	H1J1—C1J—H1J2	108.0
O3—C3—C4	119.3 (2)	O2J—C2J—C1J	108.4 (2)
O4—C3—C4	115.6 (2)	O2J—C2J—H2J1	110.0
C1E—C4—C1D	110.1 (2)	C1J—C2J—H2J1	110.0
C1E—C4—C1F	111.38 (19)	O2J—C2J—H2J2	110.0
C1D—C4—C1F	107.84 (18)	C1J—C2J—H2J2	110.0
C1E—C4—C3	105.03 (18)	H2J1—C2J—H2J2	108.4
C1D—C4—C3	110.38 (19)	C3J—O2J—C2J	113.4 (2)
C1F—C4—C3	112.12 (19)	C3J—O2J—Ba	124.66 (16)
C2D—C1D—C6D	117.8 (2)	C2J—O2J—Ba	118.34 (15)
C2D—C1D—C4	122.9 (2)	O2J—C3J—H3J1	109.5
C6D—C1D—C4	119.2 (2)	O2J—C3J—H3J2	109.5
C1D—C2D—C3D	120.9 (3)	H3J1—C3J—H3J2	109.5
C1D—C2D—H2D	119.5	O2J—C3J—H3J3	109.5
C3D—C2D—H2D	119.5	H3J1—C3J—H3J3	109.5
C4D—C3D—C2D	120.9 (3)	H3J2—C3J—H3J3	109.5
			
Ba—O1—C1—O2	31.5 (5)	O4—C3—C4—C1D	178.6 (2)
Ba—O1—C1—C2	−151.1 (3)	O3—C3—C4—C1F	−123.7 (2)
Zn—O2—C1—O1	−3.5 (3)	O4—C3—C4—C1F	58.3 (3)
Zn—O2—C1—C2	178.94 (14)	C1E—C4—C1D—C2D	11.6 (3)
O1—C1—C2—C1B	161.4 (2)	C1F—C4—C1D—C2D	−110.1 (3)
O2—C1—C2—C1B	−21.0 (3)	C3—C4—C1D—C2D	127.2 (2)
O1—C1—C2—C1A	39.8 (3)	C1E—C4—C1D—C6D	−173.1 (2)
O2—C1—C2—C1A	−142.6 (2)	C1F—C4—C1D—C6D	65.2 (3)
O1—C1—C2—C1C	−79.0 (3)	C3—C4—C1D—C6D	−57.6 (3)
O2—C1—C2—C1C	98.7 (2)	C6D—C1D—C2D—C3D	−0.9 (4)
C1B—C2—C1A—C6A	12.2 (3)	C4—C1D—C2D—C3D	174.4 (2)
C1C—C2—C1A—C6A	−109.0 (2)	C1D—C2D—C3D—C4D	0.8 (4)
C1—C2—C1A—C6A	133.8 (2)	C2D—C3D—C4D—C5D	0.1 (4)
C1B—C2—C1A—C2A	−173.5 (2)	C3D—C4D—C5D—C6D	−0.9 (4)
C1C—C2—C1A—C2A	65.3 (3)	C4D—C5D—C6D—C1D	0.8 (4)
C1—C2—C1A—C2A	−51.9 (3)	C2D—C1D—C6D—C5D	0.1 (4)
C6A—C1A—C2A—C3A	−1.0 (4)	C4—C1D—C6D—C5D	−175.4 (2)
C2—C1A—C2A—C3A	−175.6 (2)	C1D—C4—C1E—C2E	−103.2 (3)
C1A—C2A—C3A—C4A	−0.3 (4)	C1F—C4—C1E—C2E	16.4 (3)
C2A—C3A—C4A—C5A	0.9 (4)	C3—C4—C1E—C2E	138.0 (2)
C3A—C4A—C5A—C6A	−0.1 (4)	C1D—C4—C1E—C6E	74.1 (3)
C4A—C5A—C6A—C1A	−1.3 (4)	C1F—C4—C1E—C6E	−166.3 (2)
C2A—C1A—C6A—C5A	1.8 (4)	C3—C4—C1E—C6E	−44.7 (3)
C2—C1A—C6A—C5A	176.3 (2)	C6E—C1E—C2E—C3E	−0.9 (4)
C1A—C2—C1B—C2B	−108.0 (2)	C4—C1E—C2E—C3E	176.4 (2)
C1C—C2—C1B—C2B	12.0 (3)	C1E—C2E—C3E—C4E	0.3 (4)
C1—C2—C1B—C2B	128.7 (2)	C2E—C3E—C4E—C5E	0.6 (4)
C1A—C2—C1B—C6B	67.2 (3)	C3E—C4E—C5E—C6E	−0.8 (4)
C1C—C2—C1B—C6B	−172.85 (19)	C4E—C5E—C6E—C1E	0.1 (4)
C1—C2—C1B—C6B	−56.1 (3)	C2E—C1E—C6E—C5E	0.7 (4)
C6B—C1B—C2B—C3B	−0.9 (3)	C4—C1E—C6E—C5E	−176.7 (2)
C2—C1B—C2B—C3B	174.4 (2)	C1E—C4—C1F—C6F	92.9 (3)
C1B—C2B—C3B—C4B	0.0 (4)	C1D—C4—C1F—C6F	−146.2 (3)
C2B—C3B—C4B—C5B	0.6 (4)	C3—C4—C1F—C6F	−24.4 (3)
C3B—C4B—C5B—C6B	−0.3 (4)	C1E—C4—C1F—C2F	−84.8 (3)
C4B—C5B—C6B—C1B	−0.6 (4)	C1D—C4—C1F—C2F	36.1 (3)
C2B—C1B—C6B—C5B	1.1 (3)	C3—C4—C1F—C2F	157.8 (2)
C2—C1B—C6B—C5B	−174.3 (2)	C6F—C1F—C2F—C3F	−1.6 (4)
C1B—C2—C1C—C2C	−89.5 (3)	C4—C1F—C2F—C3F	176.3 (2)
C1A—C2—C1C—C2C	30.8 (3)	C1F—C2F—C3F—C4F	−0.1 (4)
C1—C2—C1C—C2C	151.7 (2)	C2F—C3F—C4F—C5F	1.8 (5)
C1B—C2—C1C—C6C	87.1 (3)	C3F—C4F—C5F—C6F	−1.9 (5)
C1A—C2—C1C—C6C	−152.6 (2)	C2F—C1F—C6F—C5F	1.5 (4)
C1—C2—C1C—C6C	−31.7 (3)	C4—C1F—C6F—C5F	−176.2 (3)
C6C—C1C—C2C—C3C	−1.3 (4)	C4F—C5F—C6F—C1F	0.2 (5)
C2—C1C—C2C—C3C	175.4 (2)	Ba—O1G—C1G—C2G	−8.8 (3)
C1C—C2C—C3C—C4C	0.4 (4)	O1G—C1G—C2G—O2G	54.1 (3)
C2C—C3C—C4C—C5C	0.5 (4)	C1G—C2G—O2G—C3G	71.7 (3)
C3C—C4C—C5C—C6C	−0.5 (4)	C1G—C2G—O2G—Ba	−67.0 (2)
C4C—C5C—C6C—C1C	−0.4 (4)	Ba—O1H—C1H—C2H	−4.1 (7)
C2C—C1C—C6C—C5C	1.2 (4)	O1H—C1H—C2H—O2H	72.7 (4)
C2—C1C—C6C—C5C	−175.4 (2)	C1H—C2H—O2H—C3H	174.4 (2)
Ba—O3—C3—O4	13.4 (5)	Ba—O1I—C1I—C2I	−140.4 (2)
Ba—O3—C3—C4	−164.39 (19)	O1I—C1I—C2I—O2I	−63.6 (3)
Zn—O4—C3—O3	22.2 (3)	C1I—C2I—O2I—C3I	105.6 (3)
Zn—O4—C3—C4	−159.98 (15)	Ba—O1J—C1J—C2J	−46.1 (3)
O3—C3—C4—C1E	115.2 (2)	O1J—C1J—C2J—O2J	56.2 (3)
O4—C3—C4—C1E	−62.8 (3)	C1J—C2J—O2J—C3J	159.6 (2)
O3—C3—C4—C1D	−3.4 (3)	C1J—C2J—O2J—Ba	−40.8 (3)

### Hydrogen-bond geometry (Å, º)

**Table:**

*D*—H···*A*	*D*—H	H···*A*	*D*···*A*	*D*—H···*A*
O1*G*—H1*G*···O2*I*^i^	0.84	1.91	2.728 (3)	163
O1*H*—H1*H*···O3	0.84	2.17	2.746 (3)	125
O1*I*—H1*I*···O2*H*	0.84	1.99	2.817 (3)	170
C2*I*—H2*I*2···Cl1^ii^	0.99	2.81	3.660 (3)	144
O1*J*—H1*J*···Cl2^iii^	0.84	2.17	3.012 (2)	174

Symmetry codes: (i) *x*−1, *y*, *z*; (ii) *x*+1, *y*, *z*; (iii) *x*, *y*+1, *z*.
